# The Genotypic Population Structure of *Mycobacterium tuberculosis* Complex from Moroccan Patients Reveals a Predominance of Euro-American Lineages

**DOI:** 10.1371/journal.pone.0047113

**Published:** 2012-10-15

**Authors:** Ouafae Lahlou, Julie Millet, Imane Chaoui, Radia Sabouni, Abdelkarim Filali-Maltouf, Mohammed Akrim, Mohammed El Mzibri, Nalin Rastogi, Rajae El Aouad

**Affiliations:** 1 National Tuberculosis Reference Laboratory, National Institute of Hygiene, Rabat, Morocco; 2 WHO Supranational TB Reference Laboratory, Unité de la Tuberculose et des Mycobactéries, Institut Pasteur de Guadeloupe, Abymes, Guadeloupe, France; 3 Biology and Medical Research Unit, National Centre of Energy, Nuclear Sciences and Techniques, Rabat, Morocco; 4 Laboratory of Microbiology and Molecular Biology, Faculty of Sciences, University of Mohammed V-Agdal, Rabat, Morocco; St. Petersburg Pasteur Institute, Russian Federation

## Abstract

**Background:**

Tuberculosis (TB) remains a major health problem in Morocco. Characterization of circulating *Mycobacterium tuberculosis* genotypic lineages, important to understand the dynamic of the disease, was hereby addressed for the first time at a national level.

**Methodology/Principal Findings:**

Spoligotyping was performed on a panel of 592 *M. tuberculosis* complex strains covering a 2-year period (2004–2006). It identified 129 patterns: 105 (n = 568 strains) corresponded to a SIT number in the SITVIT2 database, while 24 patterns were labeled as orphan. A total of 523 (88.3%) strains were clustered vs. 69 or 11.7% unclustered. Classification of strains within 3 large phylogenetical groups was as follows: group 1– ancestral/TbD1+/PGG1 (EAI, Bovis, Africanum), group 2– modern/TbD1−/PGG1 group (Beijing, CAS), group 3– evolutionary recent/TbD1−/PGG2/3 (Haarlem, X, S, T, LAM; alternatively designated as the Euro-American lineage). As opposed to group 3 strains (namely LAM, Haarlem, and T) that predominated (86.5% of all isolates), 6 strains belonged to group 2 (Beijing n = 5, CAS n = 1), and 3 strains (BOV_1 n = 2, BOV_4-CAPRAE) belonged to ancestral group 1 (EAI and AFRI lineage strains were absent). 12-loci MIRU-VNTR typing of the Casablanca subgroup (n = 114 strains) identified 71 patterns: 48 MITs and 23 orphan patterns; it allowed to reduce the clustering rate from 72.8% to 29.8% and the recent transmission rate from 64% to 20.2%.

**Conclusion:**

The *M. tuberculosis* population structure in Morocco is highly homogeneous, and is characterized by the predominance of the Euro-American lineages, namely LAM, Haarlem, and T, which belong to the “evolutionary recent” TbD1−/PGG2/3 phylogenetic group. The combination of spoligotyping and MIRUs decreased the clustering rate significantly, and should now be systematically applied in larger studies. The methods used in this study appear well suited to monitor the *M. tuberculosis* population structure for an enhanced TB management program in Morocco.

## Introduction

Tuberculosis (TB) remains an important public health problem in Morocco. The TB incidence remains high and has only slightly declined despite all the efforts to control it. The latter included the launch of a restructured TB control program in 1991 with the implementation of directly observed therapy short-course (DOTS) strategy, the availability of free diagnostics, chemotherapy, and BCG vaccination routinely applied to all infants in the first week after birth [1]. In 2010, the incidence of new cases was as high as 91/100.000 inhabitants, with a percentage of positive smear microscopy among new cases of 85% [2]. TB primarily affects young adults, with 69.5% of TB patients in the age group of 15–44 years old (72% of them being males), and therefore has a high impact on the socio-economic status of the country [3]. According to the last drug resistance survey, the prevalence of primary and secondary resistance to at least one anti-tuberculosis drug were equal to 7% and 20%, respectively, whereas, primary and secondary multidrug resistance (MDR) corresponded to 0.5% and 12% respectively [3].

In Morocco, the TB surveillance is generally performed by conventional methods based on demographic and clinical information, especially sex, age, previous TB history, close contact and microbiological confirmation tests, collected from various public health offices. This approach has limited practical value because it cannot identify the source and the route of infection and does not allow for the discrimination between reactivation cases or new infection. In this context, it is important to have a global picture on the distribution of circulating *M. tuberculosis* genotypes in Morocco. Indeed, such an epidemiological approach allows precisely characterizing predominant and emerging *M. tuberculosis* clones, leading to an improved TB control by preventing dissemination of such clones, which in turn enhances TB management by fixing the right set of priorities.

Since the discovery of polymorphic DNA sequences in *M. tuberculosis*, molecular typing has become a valuable tool in TB epidemiological studies allowing investigators to track epidemics, detect new outbreaks, achieve better knowledge of strain movement, and distinguishing between re-infection and relapse [4]. During the last decade, faster, easier to perform, robust, reliable and cost-effective techniques have been developed; the 2 most widely used PCR-based methods include spoligotyping and MIRU-VNTRs. Spoligotyping (for spacer oligonucleotide typing) is based on the analysis of the direct repeat (DR) loci, which are comprised of directly repeated sequences interspersed with variable spacer DNA [5]. MIRU-VNTR is the most promising PCR-based method which is based on the analysis of multiple loci containing Variable Numbers of Tandem Repeats (VNTR) of different families of interspersed genetic elements, collectively called Mycobacterial Interspersed Repetitive Units (MIRU); and routinely used for *M. tuberculosis* typing in 12, 15 or 24 loci formats [6], [7], [8], [9]. Although spoligotyping is frequently used as a 1^st^ line genotyping method, when used alone it overestimates the proportion of epidemiological links; a problem that may be overcome by combining it with MIRU-VNTR [10], [11].

This study was planned to characterize circulating *M. tuberculosis* complex genotypic lineages (n = 592 clinical isolates) in Morocco, using the spoligotyping technique. The subset corresponding to the city of Casablanca (n = 114) was further typed using MIRU-VNTRs to evaluate the contribution of this 2nd-line genotyping method to assess the TB transmission dynamics and the discriminatory power of the methods used. The results obtained are discussed in the context of national epidemiological surveillance and control of TB transmission.

## Results

### Patients

**Figure 1:**
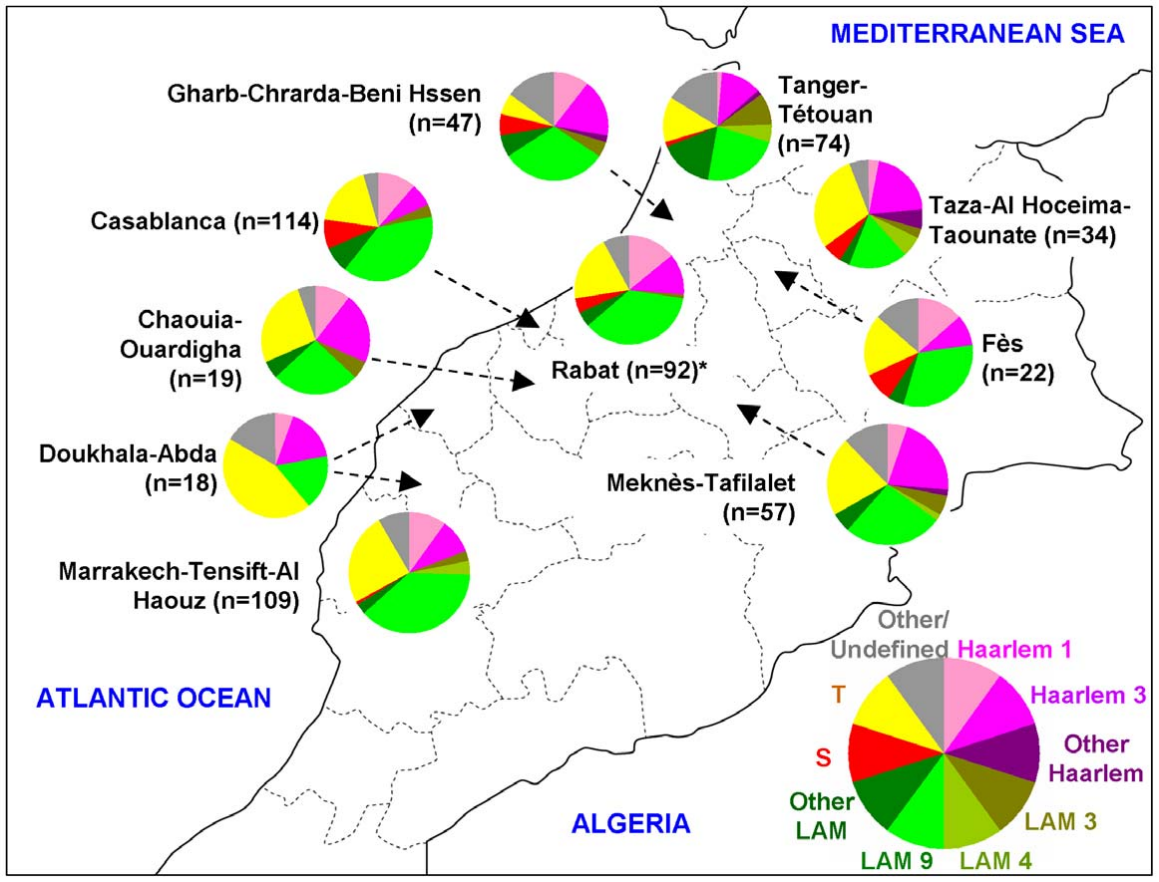
Geographical distribution of genotypic lineages of *M. tuberculosis* isolates from Morocco among ten distinct administrative regions (n = 586/592 strains; 6 strains from 3 cities were not shown as pie charts due to their low numbers: Oujda n = 1; Inezgane n = 2; Ouarzazate n = 3). Asterisk (*) denotes the administrative region of Rabat-Salé-Zemmour-Zaër). The references to color and lineages in this figure are as follows: (i) Haarlem1: SITs 47, 62, 143, 218, 609, 1139, 1155, 2333, 2567, 2 orphans; (ii) Haarlem3: SITs 36, 49, 50, 433, 741, 1135, 1539, 2338, 3077, 3078; (iii) Other Haarlem genotypes: Haarlem2 (SIT2), and Haarlem (1 orphan); (iv) LAM3: SITs 33, 105, 106; (v) LAM4: SIT60, 163, 1 orphan; (vi) LAM9: SITs 42, 161, 162, 177, 492, 731, 737, 770, 822, 866, 1070, 1074, 1075, 1154, 1277, 1832, 1 orphan; (vii) Other LAM genotypes: LAM1 (SITs 964,2372), LAM5 (SITs 93, 1072, 1999,2341), LAM6 (SIT64), LAM10-CAM (SIT61), LAM12-Madrid (SIT209), LAM (SITs 1537,3075, 7 orphans); (viii) S lineage: SIT34, 466, 784, 2516; (ix) ill-defined T: SIT7, 31, 32, 37, 40, 44, 53, 58, 65, 73, 77, 78, 102, 120,164, 244, 291, 334, 373, 751, 926, 1069, 1105, 1580,1626, 1655, 2025, 3073, 3076, 6 orphans; (v) and Other/undefined: these included Beijing (SIT1), CAS1-Delhi (SIT26), BOV_1 (SIT481, 1026), BOV_4-CAPRAE (SIT644), as well as other undefined profiles marked as Unk (unknown) in **Table S1**.

The 592 patients randomly selected for this study originated from 24 cities located in 12 of the 16 administrative regions of Morocco (**Table 1**, **Figure 1**). The demographic and epidemiological data summarized in **Table 1** showed that the age of patients ranged from 12 to 80 years (mean: 34.9 years); gender was available for 588/592 cases and corresponded to males in 72.3% of the cases (425/588), with a male to female sex-ratio of 2.61 (425/163). No significant difference between administrative regions was observed concerning distribution of age and sex-ratio. Eighty-nine percent (529/592) of the patients were new cases with significant variation between administrative regions (p<0.0001), Gharb-Chrarda-BeniHssen and Doukhala-Abda presented the lowest proportions of newly diagnosed TB cases (76.6% and 44.4% of patients respectively). The drug susceptibility testing (DST) data was available for all but 2 patients (n = 590), and showed that 91.7% (541/590) of the strains were susceptible to all the four first line drugs (i.e. isoniazid, rifampicin, ethambutol, streptomycin), while the remaining 8.3% (49/590) showed a resistance to at least one drug. Among the resistant strains, mono-resistance to rifampicin, isoniazid and streptomycin was found in 0.2%, 1.2% and 1.5% of the tested strains (n = 590) respectively, as opposed to none for ethambutol. On the other hand, MDR strains, defined as strains resistant to at least rifampicin and isoniazid, represented 1.4% of the strains (n = 8/590).

**Table 1:** Demographic and epidemiological data of the studied population from Morocco (n = 592).

Administrative regions (cities)^a^	Sex-ratio b	Mean age(years)	New casesNb (%)	Previously treated cases Nb (%)	Total Nb of cases
Gharb-Chrarda-Beni Hssen (Kenitra, Sidi Kacem)	2.9	36.6	36 (76.6) c	11 (23.4)	47
Tanger-Tetouan (Larache, Tetouan, Tanger)	2.5	31.3	69 (93.2)	5 (6.8)	74
Taza-Al Hoceima-Taounate (Al Hoceima, Taza)	2.4	34.6	30 (88.2)	4 (11.8)	34
Fes-Boulemane (Fes)	1.4	28.8	20 (90.9)	2 (9.1)	22
Meknes-Tafilalet (Meknes, Khenifra, Errachidia)	3.7	39.2	51 (89.5)	6 (10.5)	57
Rabat-Sale-Zemmour-Zaër (Rabat, Sale,Skhirat-Temara, Khemisset)	2.6	37.9	86 (93.5)	6 (6.5)	92
Grand Casablanca (Casablanca)	3.5	33.1	104 (91.2)	10 (8.8)	114
Chaouia-Ouardigha (Settat)	3.5	36.7	17 (89.5)	2 (10.5)	19
Doukhala-Abda (Safi, El Jadida)	2	29.9	8 (44.4) c	10 (55.6)	18
Marrakech-Tensift-Al Haouz (Marrakech, Essaouira)	1.9	36.4	102 (93.6)	7 (6.4)	109
L’oriental (Oujda)	NA^d^	NA^d^	1 (100)	0	1
Souss-Massa-Drâa (Inezgane, Ouarzazate)	0.7	35.0	5 (100)	0	5
Total	2.6	34.9	529	63	592

a Administrative regions (and cities) of origin for the 592 patients in the present study.

b Male to Female sex ratio.

c Significantly lower than other regions of Morocco (p<0.01).

d NA: Not Applicable since only 1 patient originated from this region.

### Spoligotyping

Spoligotyping of the 592 strains lead to 129 distinct patterns (detailed description in **Table S1**): 105 patterns (n = 568 strains) corresponded to a SIT number in the international SITVIT2 database (which is an updated version of the previous SpolDB4 database [12]; while 24 patterns corresponded to orphan profiles. With the exception of 20/129 (15.5%) patterns corresponding to 47/592 (7.9%) isolates that could not be assigned well-determined genotypic lineages in the SpolDB4 [12], or recently released SITVITWEB databases [13], and were labeled as “unknown”; the remaining 109/129 (84.5%) patterns containing 545/592 (92.1%) isolates were labeled within one of the previously described lineages. Regarding clustering, 523 strains (88.3%) were clustered (60 clusters containing 2 to 155 strains per cluster), and 69 (11.7%) were unclustered. Among the 105 SITs, 96 SITs (n = 543 isolates) corresponded to preexisting profiles in SITVIT2 whereas 9 SITs (n = 25 isolates) were newly created. Among the latter, 2 newly created SITs (SIT3072, n = 3 strains; and SIT3076, n = 7 strains) included only strains of the present study, while the remaining 7 new SITs matched preexisting orphan patterns in the SITVIT2 database as follows: SIT2836 (this study n = 1; Greece n = 1), SIT3051 (this study n = 4; Tanzania n = 1), SIT3073 (this study n = 3; USA n = 1), SIT3074 (this study n = 2; the Netherlands n = 1), SIT3075 (this study n = 3; Spain n = 1), SIT3077 (this study n = 1; Belgium n = 1) and SIT3078 (this study n = 1; Belgium n = 1). With an overall moderate discriminatory power (HGDI) of spoligotyping alone at 0.91, this typing method led to a recent transmission rate of 78.2% which is obviously an overestimation, since spoligotyping used alone tends to overestimate the proportion of clustered cases in case of highly homogeneous *M. tuberculosis* population structure [14], [15]. Indeed, the bulk of the genotypes circulating in Morocco concerned 3 families namely, LAM, Haarlem, and T (n = 512/592 or 86.5%; **Table S1**): LAM (n = 259/592 or 43.7% strains), Haarlem (n = 134/592 or 22.6% strains) and ill-defined T (n = 119/592 or 20.1% strains). Within these –3 shared-types (SIT42/LAM9, SIT53/T1, and SIT50/H3) taken together accounted for 44.7% of all TB cases studied.

Analysis of spoligotyping profiles showed that six major spoligotypes, corresponding to SIT42/LAM9, SIT53/T1, SIT50/H3, SIT47/H1, SIT33/LAM3, and SIT34/S, gathered 55.6% of the strains (**Table 2**). Worldwide geographical distribution of these 6 major SITs in the SITVIT2 database revealed their South European, American and partly African distribution. Apart the 2 common and ubiquitous profiles (SIT50, SIT53), SITs 33, 42 and 47 were predominantly distributed among European and American countries (note that SIT33 was also found in African countries); and SIT34 belonging to the modern S lineage was mainly reported among Northern American countries (almost 28% of all the SIT34 patterns in the SITVIT2 database).

**Table 2:** Description of predominant shared-types found in this study and their worldwide distribution in the SITVIT2 database.

SIT (Clade) Octal Number & Spoligotype Description	Total (%) in study	% in study vs. database	Distribution in Regions with ≥5% of a given SIT a	Distribution in countries with ≥5% of a given SIT b
33 (LAM3) 776177607760771 ▪▪▪▪▪▪▪▪□□□▪▪▪▪▪▪▪▪▪□□□□▪▪▪▪▪▪▪▪□□□□▪▪▪▪▪▪▪	16 (2.70)	1.55	AFRI-S 31.52, AMER-S 21.73, AMER-N 15.62, EURO-S 13.39, EURO-W 5.33	ZAF 31.52, USA 15.42, BRA 8.83, ESP 8.63, ARG 5.53, PER 5.43
34 (S clade) 776377777760771 ▪▪▪▪▪▪▪▪□□▪▪▪▪▪▪▪▪▪▪▪▪▪▪▪▪▪▪▪▪▪▪□□□□▪▪▪▪▪▪▪	15 (2.53)	2	AMER-N 27.96, AFRI-S 20.51, EURO-S 14.91, AMER-S 10.92, EURO-W 7.46	ZAF 20.51, USA 17.84, ITA 12.25, CAN 9.85, BRA 5.73
42 (LAM9) 777777607760771 ▪▪▪▪▪▪▪▪▪▪▪▪▪▪▪▪▪▪▪▪□□□□▪▪▪▪▪▪▪▪□□□□▪▪▪▪▪▪▪	155 (26.18)	5.62	AMER-S 28.15, AMER-N 15.36, EURO-S 12.07, AFRI-N 10.40, EURO-W 6.63	USA 14.38, BRA 9.75, MAR 8.55, COL 7.43, ITA 6.34
47 (H1) 777777774020771 ▪▪▪▪▪▪▪▪▪▪▪▪▪▪▪▪▪▪▪▪▪▪▪▪▪□□□□□□▪□□□□▪▪▪▪▪▪▪	33 (5.57)	2.67	AMER-N 20.81, EURO-W 20.00, EURO-S 13.68, AMER-S 10.36, EURO-E 8.34	USA 19.27, AUT 10.20, ITA 7.21, BRA 5.83
50 (H3) 777777777720771 ▪▪▪▪▪▪▪▪▪▪▪▪▪▪▪▪▪▪▪▪▪▪▪▪▪▪▪▪▪▪□▪□□□□▪▪▪▪▪▪▪	50 (8.45)	1.79	AMER-N 21.79, AMER-S 15.85, EURO-W 15.31, EURO-S 12.59, EURO-E 6.44, AFRI-N 5.15	USA 21.29, AUT 7.37, ESP 6.58, ITA 5.26
53 (T1) 777777777760771 ▪▪▪▪▪▪▪▪▪▪▪▪▪▪▪▪▪▪▪▪▪▪▪▪▪▪▪▪▪▪▪▪□□□□▪▪▪▪▪▪▪	60 (10.14)	1.23	AMER-N 19.37, AMER-S 14.24, EURO-W 12.62, EURO-S 9.87, ASIA-W 8.56, AFRI-S 6.40	USA 17.05, ZAF 6.26, ITA 5.05

a Worldwide distribution is reported for regions with more than 3% of a given SITs as compared to their total number in the SITVIT2 database. The definition of macro-geographical regions and sub-regions (http://unstats.un.org/unsd/methods/m49/m49regin.htm) is according to the United Nations; Regions: AFRI (Africa), AMER (Americas), ASIA (Asia), EURO (Europe), and OCE (Oceania), subdivided in: E (Eastern), M (Middle), C (Central), N (Northern), S (Southern), SE (South-Eastern), and W (Western). Furthermore, CARIB (Caribbean) belongs to Americas, while Oceania is subdivided in 4 sub-regions, AUST (Australasia), MEL (Melanesia), MIC (Micronesia), and POLY (Polynesia). Note that in our classification scheme, Russia has been attributed a new sub-region by itself (Northern Asia) instead of including it among rest of the Eastern Europe. It reflects its geographical localization as well as due to the similarity of specific TB genotypes circulating in Russia (a majority of Beijing genotypes) with those prevalent in Central, Eastern and South-Eastern Asia.

b The 3 letter country codes are according to http://en.wikipedia.org/wiki/ISO_3166-1_alpha-3; countrywide distribution is only shown for SITs with ≥5% of a given SITs as compared to their total number in the SITVIT2 database.

Geographical distribution of the *M. tuberculosis* strains within Morocco showed that the 3 most predominant SITs (SIT42, SIT53 and SIT50) were widespread in the 23 Moroccan cities included in the study (**Figure 1**). Note that 2 spoligotypes with reported phylogeographical specificity for Spain (SIT209/LAM12-Madrid1 and SIT58/T5-Madrid2) were isolated in the capital city’s administrative region of Rabat-Salé-Zemmour-Zaër (SIT209) and the cosmopolite and touristic administrative regions of Grand Casablanca, Marrakech-Tensift-Al Haouz, and Tanger-Tétouan (SIT58); n = 5 strains. Four strains with spoligotypes corresponding to the SIT61/LAM10-CAM lineage were isolated in the administration region of Taza-Al Hoceima-Taounate in the Northern part of Morocco, Grand Casablanca (which included the most important cities of Morocco, Casablanca, and Meknès-Tafilalet). Interestingly, we also found 5 (0.84%) SIT1/Beijing isolates that are characterized by hybridization only to terminal spacers 35–43; as well as a single isolate belonging to SIT26/CAS1-Delhi with reported phylogeographical specificity for Northern India [13].

### Spoligotyping-based Phylogenetical Analysis

**Figure 2:**
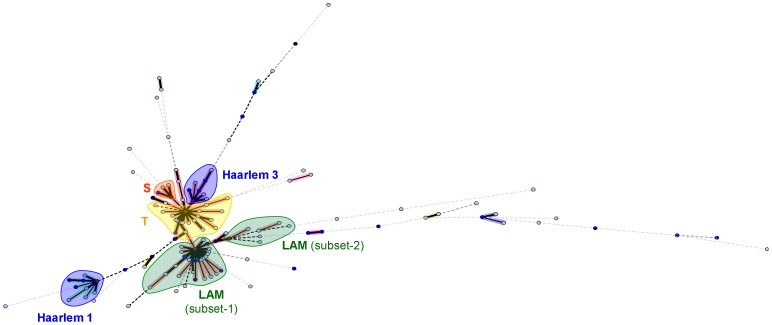
Minimum spanning tree (MST) showing evolutionary relationships among spoligotypes and lineages observed in Morocco. The length of the branches indicates the level of changes induced by loss or gain of spoligotype spacers to induce a shift from one allele to another (solid lines, single or 2 spacers changes; dotted lines, 3 or more spacer changes; color of circles shows number of isolates : sky-blue, 1 to 2 isolates; marine blue, 3 to 5 isolates; deep blue, 6 to 10 strains; brown, 11 to 20 strains; and red, >20 strains). Predominant genotypic lineages, sublineages and subsets are shown in distinct colors as follows: Haarlem (subdivided in Haarlem 1 and 3), LAM (subdivided in LAM subsets 1 and 2), S, and T (please refer to text for a detailed description).

As reviewed recently [14], the various spoligotyping-defined genotypic lineages fit well with their tentative classification in 3 large *M. tuberculosis* phylogenetical groups: ancestral TbD1+/PGG1 group (EAI, Africanum, Bovis), modern TbD1−/PGG1 group (Beijing, CAS), and evolutionary recent TbD1−/PGG2/3 group (Haarlem, X, S, T, LAM). Hence the results summarized above showed that group 3 strains namely, LAM, Haarlem, and T (corresponding to 86.5% of all isolates) predominated in our study – with the notable exception (i.e., absence) of X lineage strains supposed to be of Anglo-Saxon descent [13]. On the other hand, only 6 strains belonged to group 2 (Beijing n = 5, CAS n = 1), while 3 other strains (BOV_1 n = 2, BOV_4-CAPRAE) belonged to ancestral group 1 (note that EAI and AFRI lineage strains were absent). Subsequently, the evolutionary relationships between spoligotypes and lineages were investigated using a Minimum Spanning Tree (MST). As illustrated in **Figure 2**, five groups of strains that could shift from one to another by a single or a double spacer changes were identified (Haarlem 1, Haarlem 3, LAM subset-1, S, and T) as well as a sixth group of strains with Spoligo-profiles linked by 2 to more than 3 spacer changes (LAM subset-2) group. This MST corroborates the predominance of 3 major *M. tuberculosis* lineages in Morocco: T, LAM, and Haarlem. The central node of the Haarlem 1 group of strains (n = 51) was composed by its prototype SIT47 [13], [14], which represented 5.6% of the strains in the present study. Among the second group of Haarlem strains (i.e. the Haarlem 3 group of strains; n = 63), the central node was once again composed by its prototype SIT50 linked by only one spacer change to four other profiles and two spacer changes to the fifth one. The persistence of these distinct Haarlem sublineages in Morocco, each with a central node being represented by its own phylogenetic prototype suggests both ongoing transmission and evolution of this genotype in Morocco. Nonetheless, there is little evolution of prevailing Haarlem population of *M. tuberculosis* strains in quantitative terms since most of ongoing transmission is limited to very few predominant spoligotypes, and their diversity is almost similar to that currently observed in Euro-American countries in international databases [12], [13].

On the same line, the MST also underlined two groups of LAM strains: LAM subset-1 (n = 220) and LAM subset-2 (n = 23); LAM subset-1 was composed of 4 LAM sublineages (LAM1, LAM4, LAM5, and LAM9) while LAM subset-2 included sub-lineages SIT33/LAM3, SIT209/LAM12-Madrid, SIT1537/LAM, and 4 orphans. SIT42 (n = 155, 26.2% of strains in this study) which is the prototype of the LAM9 sublineage [13], constituted the central node of LAM subset-1; whereas SIT33 (n = 16, 2.7% of strains in this study) prototype of the LAM3 sublineage, constituted the central node of LAM subset-2 (**Figure 2**). The diversity and tight links (one or two spacer changes) observed between the 27 spoligotypes of LAM subset-1 group highlighted both ongoing transmission and evolution of this genotype in Morocco. On the other hand, the central node/SIT33 among LAM subset-2 strains was linked by more than 2 spacer changes to other profiles, including the strains belonging to LAM12-Madrid genotype as well as diverse patterns such as SIT1537/LAM and orphans. This observation and the fact that more than 2 spacer changes were required to shift from one profile to another, might suggest that this group gathered both actively transmitted SIT33/LAM3 genotype strains, as well as exogenous *M. tuberculosis* genotypes belonging to the LAM lineage that were only recently imported from Europe (and Spain in particular for the LAM12-Madrid genotype), but probably without major role in active ongoing transmission in Morocco. Lastly, two other groups were observed in the MST drawn: the T group of strains (n = 96) with 22 spoligopatterns and the condensed S group of strains (n = 23) with 4 distinct profiles. In both cases, the central nodes were made up of their prototypes (SIT53/T1, n = 60 or 10.1%; and SIT34/S, n = 16 or 2.7% of strains in study, respectively.

### MIRU Typing of the Casablanca City Sub Group

The 12-loci MIRU typing was performed on a subgroup of 114 strains isolated from patients living in Casablanca (10 spoligotyping defined clusters containing 83 strains and 31 singletons; detailed results on demographic data, drug-resistance, and genotyping information are illustrated in **Table S2**). A total of 71 distinct 12-loci MIRU profiles were obtained, and corresponded to: 57 unique profiles and 14 clusters (n = 57 strains, 2–15 strains/cluster): 1 cluster of 15 strains (MIT43), 1 cluster of 7 strains (MIT 42), 2 clusters of 5 strains (MIT347, MIT1503), 5 clusters of 3 strains (MIT38, MIT40, MIT186, MIT310, MIT680), 5 clusters of 2 strains (MIT290, MIT553, MIT998, MIT1498, MIT1504). The comparison of the MIRU profiles with patterns already reported to the SITVIT2 database led to the creation of 16 new MITs: MIT1498, MIT1499, MIT1500, MIT1501, MIT1502, MIT1503, MIT1504, MIT1505, MIT1506, MIT1507, MIT1508, MIT1509, MIT1510, MIT1511, MIT1512, and MIT1513. Three of these new MITs were created due to a match within strains from Morocco (MIT1502, 1503, 1504) while the 13 remaining MITs matched a preexisting orphan pattern from other countries (Australia, Belgium, Bulgaria, Spain, United Kingdom, USA, South Africa). Note that the 12-loci MIRU profile corresponding to MIT43 (MIRU pattern 225323153323) was the most prevalent MIRU type in our setting (n = 15/114 or 13.2%), followed by MIT42 (pattern 225313153323, n = 7/114 or 6.1%), MIT347 (pattern 224326173424, n = 5/114 or 4.4%) and MIT1503 (pattern 224324183422, n = 5/114 or 4.4%).

The combination of spoligotyping with 12-loci MIRU-VNTR profiles decreased the clustering rate from 72.8% (n = 83/114) with spoligotyping alone to 29.8% (n = 34/114). Consequently, recent transmission rate of 64% estimated on the basis of spoligotyping alone, decreased to 20.2% with the combination of spoligotyping and 12-loci MIRUs. Briefly, the 12-loci MIRU typing allowed to split the panel of 114 strains from Casablanca to a total of 91 SIT-MIT combinations among which 11 represented clusters of strains (n = 34 strains) and 80 corresponded to unique profiles within the Casablanca strain collection (**Table S2**). Among the total of 11 clusters that remained after combined spoligotyping and 12-loci MIRUs, 2 profiles were newly created (SIT42/MIT1503, n = 4 and SIT42/MIT1498, n = 2) while 9 profiles preexisted in the SITVIT2 database. The latter corresponded to SIT42/MIT42 (n = 3), SIT53/MIT310 (n = 3), SIT53/MIT40 (n = 2), SIT47/MIT43 (n = 8), SIT50/MIT43 (n = 2), SIT50/MIT290 (n = 2), SIT34/MIT680 (n = 3), SIT33/MIT186 (n = 3), and SIT218/MIT43 (n = 2). The MIT43 was found in 8/8 of SIT47 strains as well as in other shared-types (SIT62, SIT53, SIT50, SIT42 and SIT218) in following proportions: 1/1, 1/12, 2/5, 1/38, and 2/2 respectively. Indeed, clusters corresponding to SIT47, SIT33 and SIT218 remained unsplit after 12-loci MIRU-VNTR typing, whereas SIT42 (n = 38 strains) was split into 3 subclusters containing 2, 3 and 4 strains respectively, and 29 unique MIRU patterns. However, apart from the SIT47/MIT43 cluster (cluster D1; n = 8 strains) for which the male/female sex-ratio was equal to 1, no significant relationship neither to sex, age, origin, category of patients; nor drug resistance for a given SIT, MIT, lineage, and clusters could be established.

The discriminatory power (HGDI) of spoligotyping alone on the Casablanca subset (n = 114 strains) was 0.87 as compared to 0.99 for the combination spoligotyping and 12-loci MIRUs. The HGDI of different MIRU-VNTR loci summarized in **Table S3** shows that four loci with the highest diversity were MIRU10, 23, 26 and 40 that accounted for more than 67% of the total discriminatory power achieved by all 12 loci.

## Discussion

### Spoligotyping-based Genotypic Analysis

The only studies on *M. tuberculosis* molecular epidemiology and drug resistance in Morocco were attempted so far at provincial or regional level [16], [17], [18], hence knowledge on *M. tuberculosis* population structure and tuberculosis transmission was yet poorly understood at the national level. In this context, this investigation represents the first national study on the genetic diversity and the recent transmission of tuberculosis in Morocco. To characterize the population structure of *M. tuberculosis* isolates, we used spoligotyping as a first-line method to type a sample of 592 isolates from 23 cities that were selected randomly from a panel of 1300 strains analyzed within the framework of a national study on the prevalence of primary and secondary drug resistance during 2004–2006 (**Table 1**). Our results showed a high degree of diversity of genotypes with 105 SITs (n = 568 strains), among these 96 SITs containing 543 (95%) isolates matched a preexisting shared-type in the SITVIT2 database (**Table S1**). However, 9 SITs were newly created with 2 SITs specifically found in Morocco, and 7 SITs that matched an orphan *M. tuberculosis* pattern from strains isolated in 6 countries (Belgium, Greece, Tanzania, USA, Great Britain, and Spain). All the orphan patterns in our study belonged to newly detected cases of TB during the study period; however these were not associated with age of the patients (age of patients with orphan patterns varied between 19 and 65 years).

Interestingly, in spite of the high degree of spoligotype patterns observed, the population structure of *M. tuberculosis* showed remarkable homogeneity (**Table S1**, and **Table 2**). As underlined recently [13], [14], [15], spoligotyping based *M. tuberculosis* complex classification fits quite well with their overall evolutionary picture using other markers, e.g., *katG-gyrA* polymorphism [19] and the presence of a specific deletion region TbD1 [20]. The 1^st^ method permits to classify *M. tuberculosis* isolates into 3 principal genetic groups (PGG) – PGG1 strains being evolutionarily older than PGG2/3 isolates; while based on the presence or absence of the TbD1 region, the strains may be classified as ancestral (TbD1^+^) or modern (TbD1^–^). Hence, we also attempted to classify the Moroccan *M. tuberculosis* isolates in 3 large phylogenetical groups as follows: (i) group 1– ancestral TbD1^+^/PGG1 (EAI, Bovis, Africanum); (ii) group 2– modern/TbD1−/PGG1 group (Beijing, CAS); (iii) group 3– evolutionary recent/TbD1−/PGG2/3 (Haarlem, X, S, T, LAM; alternatively designated as the Euro-American lineage [21], [22]. The results obtained in this investigation showed that as opposed to group 3 strains (namely LAM, Haarlem, and T) that predominated in Morocco (86.5% of all isolates), only 6 strains belonged to group 2 (Beijing n = 5, CAS n = 1), and 3 strains belonged to ancestral group 1 (BOV_1 n = 2, BOV_4-CAPRAE). Interestingly, X lineage strains were absent among group 3 strains, while EAI and AFRI lineage strains were absent among group 1.

Overall, the majority of TB cases (83.6%) in Morocco were caused by *M. tuberculosis* belonging to the most representative clades with known phylogenetical specificity for Europe and Americas [12]: LAM (42.2% of strains) and Haarlem (22.3% of strains), as well as the ill-defined T clade (19.1% of strains). These families were split among various sublineages underlining a large and variable distribution of these in Morocco. Furthermore, each of the 3 predominant families was characterized by one or two predominant SITs, almost always representing the phylogenetic prototype of each of the lineage involved in SpolDB4 and SITVITWEB databases [12], [13]. The most predominant SIT belonged to the LAM family (SIT42, n = 155/592 or 26.2%) which represents a ubiquitous pattern belonging to the LAM9 sublineage. It was found to be widespread in all the regions of Morocco, and its predominance is most likely associated with relatively high transmission rate observed; nonetheless it remains to be seen if this predominant genotype has recently evolved due to its increased transmissibility within our population(s), or it is long established in Morocco through its stable association with respective human population [12]. Other predominant SITs included SIT50/H3 (n = 50/592 or 8.4%) and SIT47/H1 (n = 33/592 or 5.6%) of the Haarlem family, SIT53/T1 (n = 60/592 or 10.1%) of the ill-defined T family, SIT33/LAM3 (n = 16/592 or 2.7%) of the LAM family, and SIT34/S (n = 15/592 or 2.5%) belonging to the S lineage (**Table 2**).

One may recall that the ill-defined T clade (represented here T1 prototype SIT53, which is characterized by the absence of spacers 33–36) is presumed to be ancestral to the Haarlem family [21]. It is noteworthy that only the absence of spacer 31 discriminates the highly prevalent Haarlem 3 prototype SIT50/H3 from SIT53/T1, which in turn is potentially linked to IS*6110* insertional events [23]. Interestingly, a previous study from northern Tunisia showed a predominance of Haarlem H3 sublineage strains among MDR-TB patients [24]. We therefore suggest that a detailed phylogenetical study of predominant lineages showing the absence of spacers 33–36 from Morocco might help to shed light on *M. tuberculosis* evolution not only in Morocco but also within North Africa in general. Indeed, this Euro-American group of families which predominates in European and American countries [12], [13], is hereby found to be prevalent in Morocco (this study), as well as in the neighboring North African countries such as Tunisia, Algeria and Libya, making up 84.9%, 62% and 65.4% of the analyzed samples, respectively [25], [26], [27]. Considering the highly conserved population structure of these three families in Morocco and neighboring countries, as well as Europe and Americas in SpolDB4 [12], SITVITWEB [13], and SITVIT2 databases (**Table 2**), we speculate that the strains encountered were successively introduced (and reintroduced) within the Moroccan population, which is known to be composed of a mixture of people, tribes, and races with frequent travel in and outside the country for centuries [27].

Although we did not find specific spoligotypes with strong local phylogeographical specificity (**Table S1**, and **Table 2**), two spoligotypes (SIT58/T5-Madrid2 and SIT209/LAM12-Madrid1) were of particular interest; isolated from young people (20–36 years old), these could have been recently introduced to Morocco by frequent movement of people between Spain and Morocco. Another spoligotype, belonging to the SIT61/LAM10-CAM lineage (n = 4 isolates) was limited to 3 cities: Taza (n = 1), Casablanca (n = 2) and Meknes (n = 1). With phylogeographical specificity to Cameroon and neighboring countries in West Africa [12], [13], [28], the presence of these strains in Morocco can be attributed to importation by sub-Saharan migrants to and across Morocco [29]. Other interesting genotypes included six TbD1−/PGG1 group 2 strains (Beijing n = 5; CAS n = 1); both being essentially reported from different regions of Asia [12], [13], [22], [30]. Finally, strains belonging to ancestral TbD1^+^/PGG1 lineages EAI and AFRI [12], [13] were altogether absent in Morocco, with the presence of only 3 isolates belonging to *M. bovis* (BOV_1 n = 2, BOV_4-CAPRAE).

Often though not always associated with MDR-TB, Beijing genotype is an emerging pathogen in several areas with predominance in countries of East Asia [31], [32], [33], [34]. Interestingly, the 5 Beijing isolates in our study were isolated in 3 different cities (Marrakech, Fès and Sale); all were pansusceptible and their further transmission and dissemination in the population was stopped by routine TB treatment. However, any discussion around the possible introduction route(s) of these minor isolates of Beijing to Morocco remains too speculative until results of 24-loci MIRU-VNTR typing [35] and further molecular characterization [36] are interpreted in conjunction with detailed cluster and contact investigations. Lastly, the single CAS isolate in our study belonged to SIT26, which was initially reported in a study from Delhi, India [37], and later named as CAS1-Delhi [12], [13]. Whether its presence constitutes an imported case of disease (e.g. through pilgrims), remains to be investigated.

### MIRU-VNTR Typing of Casablanca City Sub Group

The MIRU-VNTR typing was performed on a subset of 114 strains from Casablanca, a metropolitan city with a mixture of population from all parts of Morocco and a high incidence of tuberculosis estimated to 160/100000 inhabitants per year [18]. As summarized in **Table S2**, the 12 loci MIRU-VNTR typing led to clustering of 57 isolates in 14 clusters. MIT43 was the most predominant pattern. It was found in 100% of SIT47/H1 (n = 8) and SIT218/H1 (n = 2) strains, in 50% of SIT50/H3 (n = 2) strains, as well as in single isolates belonging to different SITs and lineages: SIT42/LAM9, SIT53/T1, SIT62/H1. Thus despite an excellent discriminatory power of this method for all the 12-loci used (HGDI 0.974; **Table S3**), the Haarlem strains which are the 2^nd^ most prevalent lineage after LAM strains in our setting, would certainly benefit from extended MIRU typing scheme [9]. However, the combination of spoligotyping and 12-loci MIRU-VNTRs decreased the recent transmission rate from 64% on the basis of spoligotyping alone to 20.2%, showing the potential benefit of the current typing scheme in our setting. Indeed, the discriminatory power of spoligotyping used alone on the Casablanca subset (HGDI 0.87) significantly increased for the combination of spoligotyping with 12-loci MIRUs (HGDI 0.99).

Whether incidental presence of unlinked isolates in the same spoligotyping cluster may occur through coincidental reactivation of latent infections initially caused by the same phylogenetic group of *M. tuberculosis* isolates decades ago [38], [39], remains speculative in Casablanca. Nonetheless, the rate of recently transmitted disease in Morocco is lower than that estimated in other high TB incidence areas in Africa [40], [41], indirectly suggesting that reactivation of latent infection plays a more important role in persistence of disease than previously thought.

Last but not least, any discussion about “transmission” should keep in mind limitations of the typing methods and a caution in conclusions is advised seeing the homogeneity of our study sample in terms of genotypic lineages (seen through less discriminatory spoligotyping). In such a context, the classical 12-loci MIRUs might not be discriminatory enough to conclude for “recent transmission”. More discriminatory typing using the new 15-loci format for epidemiological purposes [9], [42] will therefore be needed to conclude for epi-links. Furthermore, limited patient data available in this retrospective study did not allow concluding in favor of association between genotypes/lineages observed versus occurrence of drug-resistance and type of disease (new cases, relapse, chronic tuberculosis, etc.). However, the results obtained globally justified the choice of the typing strategy used for future prospective studies in Morocco. Such prospective investigations with exhaustive epidemiologic, demographic and clinical data should allow to better understand the dynamics of the TB transmission in Morocco in order to optimize the TB control program.

### Drug Resistance Investigation

The resistance profiles to the four tested drugs of the 592 strains showed that the global resistance to any drug was 8.1% versus 1.35% for MDR (cumulative resistance to isoniazid and rifampin). These drug-resistance rates in the present set of strains corroborate data obtained in the national study indicating that *M. tuberculosis* drug resistance is relatively low in Morocco. Similar resistance rates among the unique versus clustered isolates in our study sample showed that no specific clones of resistant isolates are actively spreading in Morocco, and the lineage described did not reveal any selective advantage over the pansusceptible strains in our study. Apparently, the Direct Observed Treatment of short-course (DOTS) strategy which was introduced in Morocco in 1991 is relatively well applied.

### Conclusions

Characterization of circulating *M. tuberculosis* genotypic lineages was addressed for the first time at the national level in Morocco by spoligotyping (n = 592 strains randomly selected from a panel of 1300 isolates). It was followed by an evaluation of MIRU-VNTR minisatellites on a subset from the city of Casablanca (n = 114), as a 2nd-line genotyping method to assess the TB transmission dynamics and the discriminatory power of the methods used. The results obtained showed that the *M. tuberculosis* population structure in Morocco is characterized by the predominance of the Euro-American lineages (namely LAM, Haarlem, and T) which belong to the “evolutionary recent” TbD1−/PGG2/3 phylogenetic group. The absence of X lineage strains supposed to be of Anglo-Saxon descent [12] within the large sample studied is noteworthy, as well as the fact that ancestral group 1/TbD1+/PGG1 strains were rarely isolated; indeed, these were limited to 3 *M. bovis* strains, while no strains belonging to East-African Indian (EAI) or *M. africanum* lineages were found. Hence, the population structure from our study in Morocco was closer to the one in Europe and Americas rather than to Africa or Asia [12], [13], [43]. Interestingly, despite the phylogeographical homogeneity observed, the combination of spoligotyping and MIRUs decreased both the clustering rate (from 72.8% to 29.8%) and the recent transmission rate (from 64% to 20.2%) in Casablanca significantly. We conclude that the typing methods used in this study are well suited to monitor the *M. tuberculosis* population structure for national epidemiological surveillance and control of TB transmission, as well as an enhanced TB management program in Morocco.

## Materials and Methods

### M. tuberculosis Isolates

The present study focused on genotypic population structure of *M. tuberculosis* and only involved bacterial cultures and DNAs (n = 592 strains) randomly selected from a panel of 1300 isolates previously collected within the framework of a national study on the prevalence of primary and secondary resistance to anti-tuberculous drugs (results presented in WHO’s report number 4: anti-Tuberculosis Drug Resistance in the World; [3]). Note that sampling and testing for this earlier study were made as part of diagnosing the disease and drug resistance only for patient benefit, and no sampling or investigations were oriented by research needs. Verbal consent for diagnosis of TB and detection of drug resistance in *M. tuberculosis* isolates were obtained initially from all patients. Since the isolates were retrospectively collected from patients’ routine samples for the present study, it was considered as a laboratory study and ethics approval from institutional ethics committee was not required. It should be underlined that: (i) epidemiological and demographic data were collected using a questionnaire following informed verbal consent from patients during the previous study (see above; [3]); (ii) no studies on molecular typing of clinical isolates were yet planned at the time of sample collection, however the patients were aware that their samples could be used for TB diagnosis and research; (iii) neither this questionnaire nor the data collected were driven by the needs of the present study that was performed after the completion of the initial study, and only retrospectively; (iv) the correlation between genotypes and epidemiological and demographic data was done anonymously.

All patients were sputum microscopy positive and were included by cluster sampling from 30 cities representative of all the geographic regions and ethnic groups of Morocco, over a period of 2 years; between July 2004 and June 2006. Primary isolation and culturing of mycobacterial isolates were performed according to the procedure manual, followed by identification of *M. tuberculosis* complex using biochemical tests, including production of Niacin, Catalase activity, Nitrate reduction, pigment production and growth rate [44]. Drug susceptibility testing was performed using the 1% proportion method for Isoniazid, Rifampicin, Streptomycin and Ethambutol at the following concentrations: 0.2 µg/ml, 40 µg/ml, 4 µg/ml and 2 µg/ml respectively; multidrug resistance (MDR) was defined as resistance to at least Isoniazid and Rifampicin [44]. Epidemiological and demographic data such as age, sex, city of birth, address at the time of diagnosis, place of residence and clinical characteristics of the illness were prospectively collected using a questionnaire as part of diagnosis.

### Molecular Typing and Database Comparison

Genomic DNA was extracted by adding a loop of bacterial colonies to 200 µl of distilled water and incubating at 100°C for 10 minutes. Five µl of genomic DNA were used for PCR for genotyping with spoligotyping and 12-loci MIRU analysis. Spoligotyping was performed using a previously described protocol [5]. A subset of 114 strains from Casablanca was further typed by using the 12-loci MIRU-VNTR. The PCR-based 12-loci MIRU typing was performed using the 12-loci system and primers described previously [7], [8], [10], and the PCR products were separated by electrophoresis on a 1.5% agarose gel using an in-house high throughput protocol described recently [45].

Spoligotypes in binary format as well as the 12-loci MIRU data were entered in an Excel spreadsheet and compared to the international SITVIT2 proprietary database of the Pasteur Institute of Guadeloupe, which is an updated version of the SpolDB4 [12] and SITVITWEB [13] databases. In this database, SIT and MIT designate spoligotyping and MIRU patterns shared by 2 or more patient isolates, respectively, whereas “orphan” represents patterns reported for a single isolate. Major phylogenetic clades were assigned according to signatures provided in SpolDB4, which defines 62 genetic lineages/sublineages [12]; these include specific signatures for various *M. tuberculosis* complex members, as well as rules defining major lineages/sub-lineages for *M. tuberculosis* sensu stricto. The latter include the Beijing clade, the Central-Asian (CAS) clade and its 2 sublineages, the East-African-Indian (EAI) clade with its 9 sublineages, the Haarlem clade and its 3 sublineages, the Latin-American-Mediterranean (LAM) clade and its 12 sublineages, the “Manu” family and its 3 sublineages, the IS*6110*-low banding X clade and its 3 sublineages, and the ill-defined T clade and its 5 sublineages. Based on the spoligotyping results and clade definition, the isolates typed were also linked to the ancestral PGG1, or evolutionary younger PGG2/3 groups of *M. tuberculosis* complex as reported earlier [12], [14], [19], [20], [46].

### Statistical and Phylogenetical Analysis

Discriminatory power of a typing method (or a combination of methods) was calculated using the Hunter and Gaston Discriminatory Index [47], and significant differences between percentages were estimated using a Chi-square test. From the pattern matching, an estimate of clustering was done, and recent transmission was estimated by the formula T(c) - N(c)/T(a), where T(c) is the total number of clustered isolates, N(c) is the number of clusters, and T(a) is the total number of isolates [48].

For phylogenetical analysis, we drew minimum spanning trees based on spoligotype patterns using the BioNumerics software (Version 3.5, Applied Maths, Sint-Marteen-Latem, Belgium). MST is an undirected network in which all the samples are linked together with the smallest possible linkages between nearest neighbors. In this approach, one considers that all intermediate stages are present within the sample analyzed, by including first the individual that shows most possible linkages to other individuals within the population studied. It summarizes the phylogenetic links between two genotypes, differing by genetic changes; the length of the branches indicates the level of change required to go from one profile to another. The parameter as set did not search for hypothetical types (missing links); coefficient used was categorical, and priority rules were given to first linked types with highest number of single spacer variants.

## Supporting Information

Table S1 Description of 129 different spoligotype patterns obtained from the 592 *M. tuberculosis* clinical isolates in Morocco, followed by a comparison with the SITVIT2 database.
(PDF)

Table S2 Detailed results obtained including demographic, drug-resistance, and genotyping information on a total of 114 *M. tuberculosis* strains isolated in Casablanca, Morocco.
(PDF)

Table S3 Allelic diversity of MIRU markers observed for M. tuberculosis strains (n = 114) isolated in Casablanca.
(PDF)
