# Supplementary material for: The Genotypic Population Structure of Mycobacterium tuberculosis Complex from Moroccan Patients Reveals a Predominance of Euro-American Lineages
Source: PLoS One. 2012 Oct 15;7(10):e47113. doi: 10.1371/journal.pone.0047113 (PMC3471964; doi:10.1371/journal.pone.0047113)
Supplement: Table S1 — Description of 129 different spoligotype patterns obtained from the 592 M. tuberculosis clinical isolates in Morocco, followed by a comparison with the SITVIT2 database. (PDF) [file pone.0047113.s001.pdf]

Supplemental Table S1. Description of 129 different spoligotype patterns obtained from the 592 *M. tuberculosis* clinical isolates in Morocco, followed by a comparison with the SITVIT2 database.

SIT [a]	Spoligotype Description	Nb (%) of strains in study	% in study vs. database	Clade [b]
1	□□□□□□□□□□□□□□□□□□□□□□□□□□□□□□□□□□■■■■■■■■■	5 (0.84)	0.08	Beijing
2	□□□□□□□□□□□□□□□□□□□□□□□□■□□□□□□■□□□□■■■■■■■	4 (0.68)	1.11	H2
7	□■■■■■■■■■■■■■■■■■■■■■■■■■■■■■■■□□□□■■■■■■■	1 (0.17)	1.56	T1
26	■■■□□□□■■■■■■■■■■■■■■■□□□□□□□□□□□□■■■■■■■■■	1 (0.17)	0.1	CAS1-Delhi
31	■■■■■■■□□□□□□■■■■■■■■■■■■■■■■■■■□□□□■■■□□□□	3 (0.51)	4.84	T
32	■■■■■■■■□□□□□□□□□□□□□□□□□□□□□□□□□□□□■■■■■	1 (0.17)	1.01	T
33	■■■■■■■■□□□■■■■■■■■■□□□□■■■■■■■■□□□□■■■■■■■	16 (2.70)	1.55	LAM3
34	■■■■■■■■□□■■■■■■■■■■■■■■■■■■■■■■□□□□■■■■■■■	15 (2.53)	2	S
36	■■■■■■■■■■■■□■■■■■■■■■■■■■■■■□■□□□□■■■■■■■	3 (0.51)	3	H3
37	■■■■■■■■■■■■□■■■■■■■■■■■■■■■■■■□□□□■■■■■■■	1 (0.17)	0.31	T3
40	■■■■■■■■■■■■■■■■■■□■■■■■■■■■■■■■■□□□□■■■■■■■	2 (0.34)	1.55	T4
42	■■■■■■■■■■■■■■■■■■■■□□□□■■■■■■■■□□□□■■■■■■■	155 (26.18)	5.62	LAM9
44	■■■■■■■■■■■■■■■■■■■■■■□■■■■■■■■■■□□□□■■■■■■■	4 (0.68)	2.17	T5
47	■■■■■■■■■■■■■■■■■■■■■■■■■□□□□□□■□□□□■■■■■■■	33 (5.57)	2.67	H1
49	■■■■■■■■■■■■■■■■■■■■■■■■■■■■■■□■□□□□■■■□■■■	1 (0.17)	0.74	H3
50	■■■■■■■■■■■■■■■■■■■■■■■■■■■■■■□■□□□□■■■■■■■	50 (8.45)	1.79	H3
53	■■■■■■■■■■■■■■■■■■■■■■■■■■■■■■■■□□□□■■■■■■■	60 (10.14)	1.23	T1
58	■■■■■■■■■■■■■■■■■■■□■■□■■■■■■■■■■□□□□■■■■■■■	4 (0.68)	2.52	T5-Madrid2
60	■■■■■■■■■■■■■■■■■■■■□□□□■■■■■■■■□□□□■■■□■■■	10 (1.69)	2.72	LAM4
61	■■■■■■■■■■■■■■■■■■■■■■□□□■■■■■■■□□□□■■■■■■■	4 (0.68)	0.59	LAM10-CAM
62	■■■■■■■■■■■■■■■■■■■■■■■■■□□□□□□■□□□□■■■□■■■	4 (0.68)	0.95	H1
64	■■■■■■■■■■■■■■■■■■■■□□□□■■■■□■■■□□□□■■■■■■■	3 (0.51)	1.04	LAM6
65	■■■■■■■■■■■■■■■■■■■■■■■■■■■■■■■■□□□□■□□■■■■	1 (0.17)	1.02	T1
73	■■■■■■■■■■■■□■■■■■■■■■■■■■■■■■■□□□□■■■□■■■	1 (0.17)	0.49	T
77	■■■■□□□■■■■■■■■■■■■■■■■■■■■■■■■■□□□□■■■■■■■	2 (0.34)	9.52	T
78	■■■■■■■■■■■■■■■■■■■■■■■■■■■■■■■■□□□□■■■□□■■	2 (0.34)	3.64	T
93	■■■■■■■■■■■■□■■■■■■■■□□□□■■■■■■■■□□□□■■■■■■■	5 (0.84)	1.56	LAM5
102	■■■■■■■■■■■■□□□□■■■■■■■■■■■■■■■■□□□□■■■■■■■	1 (0.17)	1.59	T
105	■■■■■■■■□□□■■■□□□□□□□□□□□□□□□□□□□□□□□□□■■■■	2 (0.34)	4.55	LAM3
106	■■■■■■■■□□□■■■■■■■■□□□□□□□□□□□□□□□□□□□■■■■■	4 (0.68)	3.23	LAM3
120	■■■■■■■■■■■■■■■■■■■□■■■■■■■■■■■■□□□□■■■■■■■	1 (0.17)	14.29	T
143	■■■■□■■■■■□□□□□□□□□□□□□□■□□□□□□■□□□□■■■■■■■	3 (0.51)	16.67	H1
161	■■■■■■■■■■■■■■■■■■■■□□□□■■■■■■■□□□□□■■■■■■■	1 (0.17)	10	LAM9
162	■■■■■■■■■■■■■■■■■■■■□□□□■■■■■■■■□□□□■■□■■■■	1 (0.17)	4	LAM9
163	■■■■■■■■■■■■■■■■■■■■□□□□■■■■■■■■□□□□■■■□□□□	2 (0.34)	7.41	LAM4
164	■■■■■■■■■■■■■■■■■■■■■■■□□□□□□□■■□□□□■■■■■■■	3 (0.51)	6.98	T
177	□■■■■■■■■■■■■■■■■■■■□□□□■■■■■■■■□□□□■■■■■■■	1 (0.17)	1.47	LAM9
209	■■■■■■■■□□□□□□■■■■■■□□□□■■■■■■■■□□□□■■■■■■■	1 (0.17)	2.7	LAM12-Madrid1
218	■■■■■■■■■■■■□■■■■■■■■■■■□□□□□□■□□□□■■■■■■■	2 (0.34)	6.67	H1
244	■■■■■■■■■■■■■■■■■■■■■■■■■■■■■■■■□□□□■■□□□□■	1 (0.17)	0.96	T2
291	■■■■■■■■■■■■■■■■■■■■□■■■■■■■■■■■■□□□□■■■■■■■	2 (0.34)	2.63	T1
334	■□■■■■■■■■■■■■■■■■■■■■■■■■■■■■■■□□□□■■■■■■■	1 (0.17)	1.45	T1
373	■■■■■■■■■■■■■■■■■■■■■■■□■■■■■■■■□□□□■■■■■■■	1 (0.17)	1.79	T1
433	■■■■□■■■■■■■■■■■■■■■■■■■■■□■■□■□□□□■■■■■■■	10 (1.69)	23.26	H3
451	■■■■■■■■■■■■■■■■■■■□□■■■■■■■■■■■□□□□■■■■■■■	1 (0.17)	0.73	H37Rv
466	■■■■■■■■□□■■■■■■■■□■■■■■■■■■■■■■■□□□□■■■■■■■	4 (0.68)	12.5	S
481	■■□■■■■■□■■■■■■□■■■■□■■■■■■■■■■■■■■■■■□□□□□	1 (0.17)	0.2	BOV_1
492	■■■■■■□■■■■■■■■■■■■■■■□□□□■■■■■■■■□□□□■■■■■■■	2 (0.34)	11.11	LAM9
589	■■■■■■■■□□□■■■□□□■□□□□□□□□□□□□□□□□□□□□□■■■■	2 (0.34)	33.33	Unk
602	■■■■■■■■■■■■■■■■■■■■■■■■□□□□□□□□□□□□■■■■■■■	4 (0.68)	4.4	Unk
609	■■■■■■■■■■■□□□□□□□■■■■■■■□□□□□□■□□□□■■■■■■■	2 (0.34)	40	H1
644	□■□□□□□□□□□□□□□□■■□■■■■■■■■□■□□□□■■■■■□□□□□	1 (0.17)	3.45	BOV_4-CAPRAE
731	■■■■■■■■■■■■■■□□□■■■□□□□■■■■■■■■□□□□■■■■■■■	4 (0.68)	26.67	LAM9
737	■■■■■■■■■■■■■■■■■■■■□□□□■■■■■■■■□□□□■□■■■■■	1 (0.17)	7.69	LAM9
741	■■■■■■■■■■■■■■■■■■■■■■□■■■■■■■□■□□□□■■■■■■■	4 (0.68)	36.36	H3
751	□□□■■■■■■■■■■■■■■■■■■■■■■■■■■■■■□□□□■■■■■■■	1 (0.17)	7.14	T
760	■■■■■■■□□□□□□■■■■■□□□□□□□□□□□□□■□□□□■■■□□□□	1 (0.17)	11.11	Unk
770	■■■■■■■■■■■□■■■■■■■■□□□□■■■■■■■■□□□□■■■■■■■	1 (0.17)	4.76	LAM9
784	■■■■■■■■□□■■■■■■■■■■■■■■■■■■■■■■□□□□■■■□■■■	2 (0.34)	3.64	S
822	■■■■□■■■■■■■■■■■■■■■□□□□■■■■■■■■□□□□■■■■■■■	1 (0.17)	5.88	LAM9
866	■□■■■■■■■■■■■■■■■■■■□□□□■■■■■■■■□□□□■■■■■■■	6 (1.01)	14.29	LAM9
926	■■■■■■□■■■■■■■■■■■■■■■■■■■■■■■■■□□□□■■■■■■■	1 (0.17)	3.33	T1
964	■□□□■■■■■■■■■■■■■■■■□□□□■■■■■■■■□□□□■■■■■■■	6 (1.01)	40	LAM1
1026	■■□■■■■■□■■■■■■□■■■■□■■■■■■■■■■■■■□□■■□□□□□	1 (0.17)	25	BOV_1
1067	■■■■■■■□□□□□□■■■■■■■□□□■■■■■■■■■□□□□■■■□□□□	1 (0.17)	3.23	Unk

1069	■■■■■■□□■■■■■■■■■■■■■■■■■■■■■■■■□□□□■■■■■■■	1 (0.17)	4.35	T1
1070	■■■■■■■■■■■■■■■■■■■■□□□□■■■■■■■■□□□□□■■■■■■	6 (1.01)	42.86	LAM9
1072	■■■■■■■■■■■■□■■□□■■■□□□□■■■■■■■■□□□□■■■■■■■	2 (0.34)	28.57	LAM5
1074	■■■■■■■■■■■■■■■■■■■■□□□□■■■■■■■■□□□□■■■■■□■	4 (0.68)	16	LAM9
1075	■■■■■■■■■■■■■■■□□□■■□□□□■■■■■■■■□□□□■■■■■■■	1 (0.17)	16.67	LAM9
1105	■■■■■■■■■■■■■■■□■■■■■■■■■■■■■■■■□□□□■■■■■■■	2 (0.34)	9.52	T1
1135	■■■■■■■■■■■■□■■■■■■■■■■■■■■■■□■□□□□■■■□■■■	3 (0.51)	50	H3
1139	■■■■■■■■■■■■■■■■■■□■■■■■■■□□□□□□■□□□□■■■■■■■	1 (0.17)	14.29	H1
1154	■■■■■■■■■■■■■■■■■■■■□□□□■■■■■■■■□□□□■■■■□■■	1 (0.17)	7.69	LAM9
1155	■□■■■■■■■■■■■■■■■■■■■■■■■□□□□□□■□□□□■■■■■■■	1 (0.17)	11.11	H1
1277	■■■■■■■■■■■■■■■■■■□■□□□□■■■■■■■■□□□□■■■■■■■	1 (0.17)	4.76	LAM9
1536	■■■■■■■■□□■■■■■■■■■■□□□□■■■■■■■■□□□□■■■■■■■	7 (1.18)	33.33	Unk
1537	■■■■■■■■□□□■□■■■■■■■□□□□■■■■■■■■□□□□■■■□■■■	2 (0.34)	20	LAM
1538	■■■■■■■□□□□■□■■■■■■■□□□□□□□□□□□■□□□□■■■■■■■	4 (0.68)	33.33	Unk
1539	■■■■■■□■■■■■■■■■■■■■■■■■■■■■■□■□□□□■■■■■■■	1 (0.17)	6.67	H3
1541	□■■■■■■■□□■■■■■■■■■■□□□□■■■■■■■■□□□□■■■■■■■	1 (0.17)	25	Unk
1580	■■■■■■■■■■■■■■■■■■■■■■□□■■■■■■■■□□□□■■■■■■■	2 (0.34)	13.33	T
1626	■■■■■■■■■■■■■■■■■■■■■■■■■■□■■■■■■□□□□■■■■■■■	1 (0.17)	14.29	T1
1655	■■■■■■■■■■■■□■□□■■■■■■■■■■■■■■■■□□□□■■■■■■■	2 (0.34)	5.56	T3
1778	■■■■■■■■■■■■■■■□□□□□□□□□□□□□□□□□□□□□□■■■■■■	1 (0.17)	25	Unk
1832	■■■■■■■■■■■■■■■□■■■■□□□□■■■■■■■■□□□□■■■■■■■	1 (0.17)	5.56	LAM9
1999	■■■■■■■■■■■■□□□□□■■■□□□□■■■■■■■■□□□□■■■■■■■	1 (0.17)	20	LAM5
2025	■■■■■■■■■■■■■■■■■■■■■■■■■■■■■■□□□□□□□■■■■■■	1 (0.17)	12.5	T
2332	■■■■■■■□□□□□□■■■■■■■□□□□□□□■■■■■□□□□■■■□□□□	8 (1.35)	34.78	Unk
2333	■■■■■■■■■■■■■■■■■■■■■■■■■□□□□□□■□□□□■■■□□■■	3 (0.51)	30	H1
2338	■■■■■■■■■■■□□□□□□□□■■■■■■■■■■■□■□□□□■■■■■■■	2 (0.34)	33.33	H3
2341	■■■■■■■■■■■■□■■□■■■■□□□□■■■■■■■■□□□□■■■■■■■	1 (0.17)	25	LAM5
2372	■■□□□□□□□□■■■■■■■■■■□□□□■■■■■■■■□□□□■■■■■■■	1 (0.17)	33.33	LAM1
2516	■■■■□■■■□□■■■■■■■■■■■■■■■■■■■■■■□□□□■■■■■■■	2 (0.34)	40	S
2567	■■■■■■■■■■■■■■■□□■□■■■■■■□□□□□□■□□□□■■■■■■■	2 (0.34)	50	H1
2836*	□□□□□□□□□□□□□□□□□■■■■■■■■■■■■■■■□□□□■■■■■■■	1 (0.17)	50	Unk
2891	■■■■■■■■■■■■■■■□■■□□□□□□□□□□□□□□□□□□□■■□■■■	2 (0.34)	50	Unk
3051*	■■■■■■■■■■■■■■■□□■□□□□□□□□□□□□□□□□□□□■■■■■■	4 (0.68)	80	Unk
3072*	■■■■■■■□□□□■□■■■■■■■□□□□□□□□□□■■□□□□■■■■■■■	3 (0.51)	100	Unk
3073*	■■■■■■■■■■□□□■■■■■■■■■■■■■■■■■■■□□□□■■■□□□□	3 (0.51)	75	T
3074*	■■■■■■□■■■□□□□■■■■■■■■■□□□□□□□■■□□□□■■■■■■■	2 (0.34)	66.67	Unk
3075*	■■■■■■■■■■■□□□■□■■■□□□□□■□■■■■■■□□□□■□□■■■■	3 (0.51)	75	LAM
3076*	■■■■■■■■■■■■■■■■■■■■■■□□□■□□□■■■□□□□■■■■■■■	7 (1.18)	100	T
3077*	■□■□□□□□□□■■■■■■■■■■■■■■■■■■■■□■□□□□■■■■■■■	1 (0.17)	50	H3
3078*	■■■■■■■□□□□□□■■■■■■■■■■■■■■■■■□■□□□□■■■■■■■	1 (0.17)	50	H3
Orphan1	□□□□□□□□□□□□□□□□□□□□□□□□■■■■■■■□□□□□□□□■■■■	1 (0.17)	–	Unk
Orphan2	□■■■□■■■■■■■■■■■■■■■□□□□■■■■■■■■□□□□■□■■■■■	1 (0.17)	–	LAM
Orphan3	■■□□□□□□□□□□□□□□■■■■□□□□■■■■■■■■□□□□■■■■■■■	1 (0.17)	–	LAM
Orphan4	■■■□■■■■■□■■■■■■■■■■□□□□■■■■■■■■□□□□■■■■■□■	1 (0.17)	–	LAM9
Orphan5	■■■□■■■■■■■■■■■■■■■■□□□□■■■■■■■■□□□□■■■□■■■	1 (0.17)	–	LAM4
Orphan6	■■■■□■■■■■■■■■■■■■■■■■■■■■□■■■■□□□□■■■■■■■	1 (0.17)	–	T
Orphan7	■■■■■□□□□□□□□□■■■■■■□□□□■■■■■■■■□□□□■■■■■■■	1 (0.17)	–	LAM
Orphan8	■■■■■□■■■■■■■■■■■■■■■■■■■■■■■■■■□□□□■■□□■■■	1 (0.17)	–	T
Orphan9	■■■■■■□■■■■■■■■■■■■■□■■■■□□□□□□■□□□□■■■■■■■	1 (0.17)	–	H1
Orphan10	■■■■■■■□□□□□□■■■■■□■■■■■■■■■■■■■□□□□■■■□□□□	1 (0.17)	–	T
Orphan11	■■■■■■■□□□□■□■■■■■■■□□□□■■■■□■■■□□□□■■■■■■■	1 (0.17)	–	LAM
Orphan12	■■■■■■■■□□□□□■■■■■■■□□□□■■□■■■■■□□□□■■■■■■■	1 (0.17)	–	LAM
Orphan13	■■■■■■■■□□□■■■■■■■■■□□□□■■■■■■■□□□□□■■■□□■■	1 (0.17)	–	LAM
Orphan14	■■■■■■■■■□□□□□□□□■■■■■□□■□■■■■□■□□□□■■■■■■■	1 (0.17)	–	Unk
Orphan15	■■■■■■■■■□□□□□□□□■■■■■□□■□■■■■■■□□□□■■■■■■■	1 (0.17)	–	Unk
Orphan16	■■■■■■■■■■■■□□□□□■□□□□□■■■■■■■■■□□□□■■■■■■■	1 (0.17)	–	T
Orphan17	■■■■■■■■■■■■□■■■■■■■■■■■■■■■■□■■□□□□■■■□□□□	1 (0.17)	–	T
Orphan18	■■■■■■■■■■■■■□□□□■□□□□□□□□□□□□□□□□□□□□□□□□□	1 (0.17)	–	Unk
Orphan19	■■■■■■■■■■■■■□□□□■□□□□□■■□□□□□□■□□□□■■■■■■■	1 (0.17)	–	H1
Orphan20	■■■■■■■■■■■■■■■■■■□□□□□□■□■■■■■■□□□□■□□■■■■	1 (0.17)	–	Unk
Orphan21	■■■■■■■■■■■■■■■■■■□□□■■■■■■■□□□□□□□□□□□□□□■	1 (0.17)	–	Unk
Orphan22	■■■■■■■■■■■■■■■■■■■■□□□□■■■□■■■■□□□□■□□□■□■	1 (0.17)	–	LAM
Orphan23	■■■■■■■■■■■■■■■■■■■■□□■■■■■■■■■■□□□□■■■□■■■	1 (0.17)	–	T
Orphan24	■■■■■■■■■■■■■■■■■■■■■■□□□■□□□■□■□□□□■■■■■■■	1 (0.17)	–	H

[a] In this study, 129 distinct spoligotype patterns were obtained from 592 *M. tuberculosis* isolates; 523/592 (88.3%) were clustered (60 clusters, 2-155 strains/cluster), and 69 (11.7%) were unclustered. A total of 96 SITs containing 543 isolates matched a pre-existing shared type in SITVIT2, whereas 9 SITs (containing 25 Isolates) were newly-created either within the present study or after a match with an orphan in the database (showed by an asterisk; SIT2836, SIT3051,SIT3072, SIT3073, SIT3074, SIT3075, SIT3076, SIT3077, SIT3078).

[b] Clade designations according to SITVIT2 using revised SpolDB4 rules; Unk: Unknown pattern within any of the major clades described in SITVIT2 database.

[a] In this study, 129 distinct spoligotype patterns were obtained from 592 *M. tuberculosis* isolates; 523/592 (88.3%) were clustered (60 clusters, 2-155 strains/cluster), and 69 (11.7%) were unclustered. A total of 96 SITs containing 543 isolates matched a pre-existing shared type in SITVIT2, whereas 9 SITs (containing 25 Isolates) were newly-created either within the present study or after a match with an orphan in the database (showed by an asterisk; SIT2836, SIT3051,SIT3072, SIT3073, SIT3074, SIT3075, SIT3076, SIT3077, SIT3078).

[b] Clade designations according to SITVIT2 using revised SpolDB4 rules; Unk: Unknown pattern within any of the major clades described in SITVIT2 database
