# Supplementary material for: The Genotypic Population Structure of Mycobacterium tuberculosis Complex from Moroccan Patients Reveals a Predominance of Euro-American Lineages
Source: PLoS One. 2012 Oct 15;7(10):e47113. doi: 10.1371/journal.pone.0047113 (PMC3471964; doi:10.1371/journal.pone.0047113)
Supplement: Table S2 — Detailed results obtained including demographic, drug-resistance, and genotyping information on a total of 114 M. tuberculosis strains isolated in Casablanca, Morocco. (PDF) [file pone.0047113.s002.pdf]

<u>Supplemental Table S2</u>. Detailed results obtained including demographic, drug-resistance, and genotyping information on a total of 114 *M. tuberculosis* strains isolated in Casablanca, Morocco.

Strain	Year	Sex/Age	DST[a]	Drugs[b]	Binary Spoligotype	SIT	Clade[c]	Remarks	MIRU12	MIT	Conclusion
0443	2004	M/26	1		■■■■■■■■□□□■■■■■■■■■□□□□■■■■■■■■□□□□■■■■■■■	33	LAM3		224226153314	186	
0708	2005	F/45	1		■■■■■■■■□□□■■■■■■■■■□□□□■■■■■■■■□□□□■■■■■■■	33	LAM3	Cluster A	224226153314	186	Cluster A1
1405	2005	M/40	1		■■■■■■■■□□□■■■■■■■■■□□□□■■■■■■■■□□□□■■■■■■■	33	LAM3		224226153314	186	
0540	2004	M/18	1		■■■■■■■■□□■■■■■■■■■■■■■■■■■■■■■■□□□□■■■■■■■	34	S		225313153323	42	
0763	2004	M/24	1		■■■■■■■■□□■■■■■■■■■■■■■■■■■■■■■■□□□□■■■■■■■	34	S		333315153222	185	Unclustered
0605	2005	M/36	1		■■■■■■■■□□■■■■■■■■■■■■■■■■■■■■■■□□□□■■■■■■■	34	S		333325153222	279	
0603	2005	M/29	1		■■■■■■■■□□■■■■■■■■■■■■■■■■■■■■■■□□□□■■■■■■■	34	S		333215153222	680	
0611	2004	M/28	1		■■■■■■■■□□■■■■■■■■■■■■■■■■■■■■■■□□□□■■■■■■■	34	S	Cluster B	333215153222	680	Cluster B1
0774	2004	F/21	1		■■■■■■■■□□■■■■■■■■■■■■■■■■■■■■■■□□□□■■■■■■■	34	S		333215153222	680	
0709	2004	F/45	1		■■■■■■■■□□■■■■■■■■■■■■■■■■■■■■■■□□□□■■■■■■■	34	S		333315153322	1354	
0477	2004	M/53	1		■■■■■■■■□□■■■■■■■■■■■■■■■■■■■■■■□□□□■■■■■■■	34	S		333215153223	Orphan	Unclustered
0770	2004	F/75	1		■■■■■■■■□□■■■■■■■■■■■■■■■■■■■■■■□□□□■■■■■■■	34	S		333215153322	Orphan	
1001	2005	M/26	1		■■■■■■■■■■■■■■■■■■■■□□□□■■■■■■■■□□□□■■■■■■■	42	LAM9		223222153323	9	Unclustered
0399	2004	F/36	1		■■■■■■■■■■■■■■■■■■■■□□□□■■■■■■■■□□□□■■■■■■■	42	LAM9		224326143323	38	
0726	2005	M/46	1		■■■■■■■■■■■■■■■■■■■■□□□□■■■■■■■■□□□□■■■■■■■	42	LAM9		225313153323	42	
0764	2004	M/38	1		■■■■■■■■■■■■■■■■■■■■□□□□■■■■■■■■□□□□■■■■■■■	42	LAM9		225313153323	42	Cluster C1
0765	2004	F/24	1		■■■■■■■■■■■■■■■■■■■■□□□□■■■■■■■■□□□□■■■■■■■	42	LAM9		225313153323	42	
0707	2005	M/48	1		■■■■■■■■■■■■■■■■■■■■□□□□■■■■■■■■□□□□■■■■■■■	42	LAM9		225323153323	43	
0717	2005	M/48	1		■■■■■■■■■■■■■■■■■■■■□□□□■■■■■■■■□□□□■■■■■■■	42	LAM9		225313153322	184	
0720	2005	M/19	1		■■■■■■■■■■■■■■■■■■■■□□□□■■■■■■■■□□□□■■■■■■■	42	LAM9		224326173424	347	
0410	2004	F/39	1		■■■■■■■■■■■■■■■■■■■■□□□□■■■■■■■■□□□□■■■■■■■	42	LAM9		224226133321	464	
0718	2005	M/47	1		■■■■■■■■■■■■■■■■■■■■□□□□■■■■■■■■□□□□■■■■■■■	42	LAM9		223315153324	553	Unclustered
1008	2005	M/33	1		■■■■■■■■■■■■■■■■■■■■□□□□■■■■■■■■□□□□■■■■■■■	42	LAM9		224316143323	998	
0752	2004	M/45	1		■■■■■■■■■■■■■■■■■■■■□□□□■■■■■■■■□□□□■■■■■■■	42	LAM9		124327153326	1237	
1010	2005	F/24	1		■■■■■■■■■■■■■■■■■■■■□□□□■■■■■■■■□□□□■■■■■■■	42	LAM9		124327153324	1241	
0741	2004	M/44	1		■■■■■■■■■■■■■■■■■■■■□□□□■■■■■■■■□□□□■■■■■■■	42	LAM9		124226153324	1248	
0396	2004	M/31	1		■■■■■■■■■■■■■■■■■■■■□□□□■■■■■■■■□□□□■■■■■■■	42	LAM9		124326143326	1498	Cluster C2
0740	2005	M/21	1		■■■■■■■■■■■■■■■■■■■■□□□□■■■■■■■■□□□□■■■■■■■	42	LAM9		124326143326	1498	
0433	2004	M/22	1		■■■■■■■■■■■■■■■■■■■■□□□□■■■■■■■■□□□□■■■■■■■	42	LAM9		224326133424	1499	
0444	2004	M/18	1		■■■■■■■■■■■■■■■■■■■■□□□□■■■■■■■■□□□□■■■■■■■	42	LAM9		223326163424	1500	Unclustered
0760	2004	M/36	1		■■■■■■■■■■■■■■■■■■■■□□□□■■■■■■■■□□□□■■■■■■■	42	LAM9	Cluster C	225225182524	1501	
0422	2004	M/40	1		■■■■■■■■■■■■■■■■■■■■□□□□■■■■■■■■□□□□■■■■■■■	42	LAM9		224324173422	1502	
0476	2004	M/45	1		■■■■■■■■■■■■■■■■■■■■□□□□■■■■■■■■□□□□■■■■■■■	42	LAM9		224324183422	1503	
0631	2005	F/28	1		■■■■■■■■■■■■■■■■■■■■□□□□■■■■■■■■□□□□■■■■■■■	42	LAM9		224324183422	1503	Cluster C3
0725	2005	M/26	1		■■■■■■■■■■■■■■■■■■■■□□□□■■■■■■■■□□□□■■■■■■■	42	LAM9		224324183422	1503	
0738	2005	M/41	1		■■■■■■■■■■■■■■■■■■■■□□□□■■■■■■■■□□□□■■■■■■■	42	LAM9		224324183422	1503	
1015	2005	M/21	3	S,I	■■■■■■■■■■■■■■■■■■■■□□□□■■■■■■■■□□□□■■■■■■■	42	LAM9		228324163423	1504	
0628	2004	M/73	1		■■■■■■■■■■■■■■■■■■■■□□□□■■■■■■■■□□□□■■■■■■■	42	LAM9		224326173324	1505	
0384	2004	M/24	2	S,I,R,E	■■■■■■■■■■■■■■■■■■■■□□□□■■■■■■■■□□□□■■■■■■■	42	LAM9		224326162224	Orphan	
0478	2005	M/22	1		■■■■■■■■■■■■■■■■■■■■□□□□■■■■■■■■□□□□■■■■■■■	42	LAM9		123327183427	Orphan	
0485	2004	M/35	1		■■■■■■■■■■■■■■■■■■■■□□□□■■■■■■■■□□□□■■■■■■■	42	LAM9		243326143321	Orphan	
0591	2004	M/28	3	S	■■■■■■■■■■■■■■■■■■■■□□□□■■■■■■■■□□□□■■■■■■■	42	LAM9		124325153328	Orphan	
0600	2005	M/20	1		■■■■■■■■■■■■■■■■■■■■□□□□■■■■■■■■□□□□■■■■■■■	42	LAM9		224326173422	Orphan	
0609	2005	F/26	1		■■■■■■■■■■■■■■■■■■■■□□□□■■■■■■■■□□□□■■■■■■■	42	LAM9		124327153226	Orphan	Unclustered
0706	2005	F/37	1		■■■■■■■■■■■■■■■■■■■■□□□□■■■■■■■■□□□□■■■■■■■	42	LAM9		222324163422	Orphan	
0722	2005	M/20	1		■■■■■■■■■■■■■■■■■■■■□□□□■■■■■■■■□□□□■■■■■■■	42	LAM9		224326163424	Orphan	
0750	2005	M/41	1		■■■■■■■■■■■■■■■■■■■■□□□□■■■■■■■■□□□□■■■■■■■	42	LAM9		224325183422	Orphan	
0761	2004	M/39	1		■■■■■■■■■■■■■■■■■■■■□□□□■■■■■■■■□□□□■■■■■■■	42	LAM9		124326163327	Orphan	
1003	2005	F/17	1		■■■■■■■■■■■■■■■■■■■■□□□□■■■■■■■■□□□□■■■■■■■	42	LAM9		224326183424	Orphan	
1401	2005	F/25	1		■■■■■■■■■■■■■■■■■■■■□□□□■■■■■■■■□□□□■■■■■■■	42	LAM9		234323163323	Orphan	

0739	2005	M/25	3	S,I	■■■■■■■■■■■■■■■■■■■■■■□■■■■■■■■■■□□□□■■■■■■■	44	T5	Unclustered	223315153324	553	Unclustered
0326	2004	M/19	1		■■■■■■■■■■■■■■■■■■■■■■■■■□□□□□□■□□□□■■■■■■■	47	H1		225323153323	43	
0327	2004	M/27	1		■■■■■■■■■■■■■■■■■■■■■■■■■□□□□□□■□□□□■■■■■■■	47	H1		225323153323	43	
0357	2004	M/38	1		■■■■■■■■■■■■■■■■■■■■■■■■■□□□□□□■□□□□■■■■■■■	47	H1		225323153323	43	
0388	2004	M/58	1		■■■■■■■■■■■■■■■■■■■■■■■■■□□□□□□■□□□□■■■■■■■	47	H1	Cluster D	225323153323	43	Cluster D1
0407	2006	M/31	1		■■■■■■■■■■■■■■■■■■■■■■■■■□□□□□□■□□□□■■■■■■■	47	H1		225323153323	43	
0606	2005	M/23	1		■■■■■■■■■■■■■■■■■■■■■■■■■□□□□□□■□□□□■■■■■■■	47	H1		225323153323	43	
0634	2004	F/38	1		■■■■■■■■■■■■■■■■■■■■■■■■■□□□□□□■□□□□■■■■■■■	47	H1		225323153323	43	
0732	2005	M/21	1		■■■■■■■■■■■■■■■■■■■■■■■■■□□□□□□■□□□□■■■■■■■	47	H1		225323153323	43	
0587	2005	M/32	1		■■■■■■■■■■■■■■■■■■■■■■■■■■■■■■□■□□□□■■■■■■■	50	H3		225323153323	43	Cluster E1
0607	2004	M/31	1		■■■■■■■■■■■■■■■■■■■■■■■■■■■■■■□■□□□□■■■■■■■	50	H3		225323153323	43	
0766	2005	M/20	1		■■■■■■■■■■■■■■■■■■■■■■■■■■■■■■□■□□□□■■■■■■■	50	H3	Cluster E	226313153323	290	Cluster E2
0767	2004	M/47	3	S,R	■■■■■■■■■■■■■■■■■■■■■■■■■■■■■■□■□□□□■■■■■■■	50	H3		226313153323	290	
0417	2004	M/26	1		■■■■■■■■■■■■■■■■■■■■■■■■■■■■■■□■□□□□■■■■■■■	50	H3		224326173424	347	Unclustered
1002	2005	F/19	1		■■■■■■■■■■■■■■■■■■■■■■■■■■■■■■■■□□□□■■■■■■■	53	T1		224315153323	27	Unclustered
0440	2005	M/27	1		■■■■■■■■■■■■■■■■■■■■■■■■■■■■■■■■□□□□■■■■■■■	53	T1		225125113322	40	Cluster F1
0771	2005	M/40	1		■■■■■■■■■■■■■■■■■■■■■■■■■■■■■■■■□□□□■■■■■■■	53	T1		225125113322	40	
0602	2005	F/75	1		■■■■■■■■■■■■■■■■■■■■■■■■■■■■■■■■□□□□■■■■■■■	53	T1		225323153323	43	Unclustered
0450	2004	M/34	1		■■■■■■■■■■■■■■■■■■■■■■■■■■■■■■■■□□□□■■■■■■■	53	T1		215125113322	310	
0719	2005	M/30	1		■■■■■■■■■■■■■■■■■■■■■■■■■■■■■■■■□□□□■■■■■■■	53	T1	Cluster F	215125113322	310	Cluster F2
0778	2005	M/34	1		■■■■■■■■■■■■■■■■■■■■■■■■■■■■■■■■□□□□■■■■■■■	53	T1		215125113322	310	
0622	2004	F/26	1		■■■■■■■■■■■■■■■■■■■■■■■■■■■■■■■■□□□□■■■■■■■	53	T1		224326173424	347	
0341	2004	F/28	1		■■■■■■■■■■■■■■■■■■■■■■■■■■■■■■■■□□□□■■■■■■■	53	T1		228325163423	415	
1007	2005	F/38	1		■■■■■■■■■■■■■■■■■■■■■■■■■■■■■■■■□□□□■■■■■■■	53	T1		224316143323	998	Unclustered
1085	2005	M/34	2	I,R	■■■■■■■■■■■■■■■■■■■■■■■■■■■■■■■■□□□□■■■■■■■	53	T1		228324163423	1504	
0601	2005	M/30	1		■■■■■■■■■■■■■■■■■■■■■■■■■■■■■■■■□□□□■■■■■■■	53	T1		243226143321	1506	
0424	2004	M/31	1		■■■■■■■■■■■■■■■■■■■□■■□■■■■■■■■■■□□□□■■■■■■■	58	T5-Madrid2	Unclustered	223326153324	18	Unclustered
0398	2004	M/21	1		■■■■■■■■■■■■■■■■■■■■■■□□□■■■■■■■□□□□■■■■■■■	61	LAM10-CAM	Cluster G	223315153323	12	Unclustered
0404	2004	M/43	1		■■■■■■■■■■■■■■■■■■■■■■□□□■■■■■■■□□□□■■■■■■■	61	LAM10-CAM		224226173424	Orphan	
0597	2004	M/28	1		■■■■■■■■■■■■■■■■■■■■■■■■■□□□□□□■□□□□■■■□■■■	62	H1	Unclustered	225323153323	43	Unclustered
0461	2004	M/25	1		■■■■■■■■■■■■□■■■■■■■■■■■■■■■■■■■□□□□■■■□■■■	73	T	Unclustered	225313153323	42	Unclustered
0734	2004	M/24	1		■■■■■■■■■■■■■■■■■■■■■■■■■■■■■■■■□□□□■■■□□■■	78	T	Unclustered	225125113322	40	Unclustered
0781	2005	F/45	1		■■■■■■■■□□□■■■■■■■■□□□□□□□□□□□□□□□□□□□■■■■■	106	LAM3	Unclustered	224326133324	313	Unclustered
0633	2004	M/38	1		■■■■□■■■■■□□□□□□□□□□□□□□■□□□□□□■□□□□■■■■■■■	143	H1	Unclustered	224215153323	Orphan	Unclustered
0735	2004	M/55	1		■■■■■■■■■■■■■■■■■■■■□□□□■■■■■■■■□□□□■■■□□□□	163	LAM4	Unclustered	124226153327	Orphan	Unclustered
0721	2005	M/37	1		■■■■■■■■■■■■■■■■■■■■■■■□□□□□□□■■□□□□■■■■■■■	164	T	Unclustered	124327163326	Orphan	Unclustered
0467	2004	M/60	1		□■■■■■■■■■■■■■■■■■■■□□□□■■■■■■■■□□□□■■■■■■■	177	LAM9	Unclustered	224325163423	1507	Unclustered
0436	2004	M/44	1		■■■■■■■■■■■■□■■■■■■■■■■■■□□□□□□■□□□□■■■■■■■	218	H1	Cluster H	225323153323	43	Cluster H1
0620	2005	M/33	3	R	■■■■■■■■■■■■□■■■■■■■■■■■■□□□□□□■□□□□■■■■■■■	218	H1		225323153323	43	
0730	2005	M/24	1		■■■■□■■■■■■■■■■■■■■■■■■■■■■□■■□■□□□□■■■■■■■	433	H3	Cluster I	225324153323	740	Unclustered
0769	2004	F/42	1		■■■■□■■■■■■■■■■■■■■■■■■■■■■□■■□■□□□□■■■■■■■	433	H3		223316153323	Orphan	
0753	2004	F/27	3	S,I	■■■■■■□■■■■■■■■■■■■■□□□□■■■■■■■■□□□□■■■■■■■	492	LAM9	Unclustered	224325182424	345	Unclustered
0616	2004	M/30	3	S	■■■■■■■■■■■■■■□□□■■■□□□□■■■■■■■■□□□□■■■■■■■	731	LAM9	Unclustered	224326123321	Orphan	Unclustered
0466	2004	M/30	1		■■■■■■■■■■■□■■■■■■■■□□□□■■■■■■■■□□□□■■■■■■■	770	LAM9	Unclustered	224325162423	1508	Unclustered
0369	2004	M/24	1		■□■■■■■■■■■■■■■■■■■■□□□□■■■■■■■■□□□□■■■■■■■	866	LAM9	Unclustered	224326163325	1509	Unclustered
0319	2004	F/19	1		■□□□■■■■■■■■■■■■■■■■□□□□■■■■■■■■□□□□■■■■■■■	964	LAM1	Unclustered	224326173424	347	Unclustered
0630	2004	M/34	1		■■■■■■■■■■■■□■■□□■■■□□□□■■■■■■■■□□□□■■■■■■■	1072	LAM5	Unclustered	224326143323	38	Unclustered
0400	2005	M/42	1		■■■■■■■■■■■■■■■□□□■■□□□□■■■■■■■■□□□□■■■■■■■	1075	LAM9	Unclustered	224326143323	38	Unclustered
0585	2005	M/27	1		■■■■■■■■■■■■□■■■■■■■■■■■■■■■■■□■□□□□■■■□■■■	1135	H3	Unclustered	225313153323	42	Unclustered
0727	2005	M/21	1		■■■■■■■■□□□■□■■■■■■■□□□□■■■■■■■■□□□□■■■□■■■	1537	LAM	Unclustered	124327153325	1510	Unclustered
0479	2004	M/56	1		■■■■■■■■■■■■■■■■■■■■■■□□■■■■■■■■□□□□■■■■■■■	1580	T	Unclustered	123315153323	Orphan	Unclustered
1400	2005	M/35	3	S,I	■■■■■■■■■■■■□■□□■■■■■■■■■■■■■■■■□□□□■■■■■■■	1655	T3	Unclustered	238321153323	Orphan	Unclustered
0392	2004	F/28	1		■■■■■■■□□□□□□■■■■■■■□□□□□□□■■■■■□□□□■■■□□□□	2332	Unk	Unclustered	223325151323	1511	Unclustered
0434	2005	M/19	1		■■■■■■■■■■■■■■■■■■■■■■■■■□□□□□□■□□□□■■■□□■■	2333	H1	Unclustered	225323153423	1104	Unclustered
1014	2005	M/21	1		■■■■□■■■□□■■■■■■■■■■■■■■■■■■■■■■□□□□■■■■■■■	2516	S	Unclustered	225313153323	42	Unclustered

0736	2004	M/25	3	S	□□□□□□□□□□□□□□□□□■■■■■■■■■■■■■■■□□□□■■■■■■■	2836	Unk	Unclustered	333325153324	397	Unclustered
0408	2004	F/19	1		■■■■■■■■■■■■■■■□□■□□□□□□□□□□□□□□□□□□□■■■■■■	3051	Unk	Cluster J	224324183422	1503	Unclustered
0414	2004	M/27	1		■■■■■■■■■■■■■■■□□■□□□□□□□□□□□□□□□□□□□■■■■■■	3051	Unk		233323153323	1512	Unclustered
0705	2005	M/34	1		■■■■■■■□□□□■□■■■■■■■□□□□□□□□□□■■□□□□■■■■■■■	3072	Unk	Unclustered	224326173424	347	Unclustered
0737	2004	M/36	1		■■■■■■■■■■□□□■■■■■■■■■■■■■■■■■■■□□□□■■■□□□□	3073	T	Unclustered	223325153325	219	Unclustered
0475	2004	M/29	1		■■■■■■■□□□□■□■■■■■■■□□□□■■■■□■■■□□□□■■■■■■■	Orphan11	LAM	Unclustered	224325153322	32	Unclustered
0716	2005	M/31	2	S,I,R	□■■■□■■■■■■■■■■■■■■■□□□□■■■■■■■■□□□□■□■■■■■	Orphan2	LAM	Unclustered	224326163321	375	Unclustered
0481	2005	F/29	1		■■■■□■■■■■■■■■■■■■■■■■■■■■■□■■■■□□□□■■■■■■■	Orphan6	T	Unclustered	224323153324	1513	Unclustered
1005	2005	M/40	1		■■□□□□□□□□□□□□□□■■■■□□□□■■■■■■■■□□□□■■■■■■■	Orphan3	LAM	Unclustered	124326163325	Orphan	Unclustered

[a] DST, Drug Susceptibility Testing: 0, information not available in database; 1, pansusceptible; 2, multidrug-resistant (isoniazid + rifampin), 3, any resistance.
[b] Drugs, indicates the profile of resistance if any resistance has been detected: S, Streptomycin; I, Isoniazid; R, Rifampicin; E, Ethambutol.
[c] Clade designations according to SITVIT2 using revised SpolDB4 rules; Unk: Unknown pattern within any of the major clades described in SITVIT2 database
