# Supplementary material for: The Genotypic Population Structure of Mycobacterium tuberculosis Complex from Moroccan Patients Reveals a Predominance of Euro-American Lineages
Source: PLoS One. 2012 Oct 15;7(10):e47113. doi: 10.1371/journal.pone.0047113 (PMC3471964; doi:10.1371/journal.pone.0047113)
Supplement: Table S3 — Allelic diversity of MIRU markers observed for M. tuberculosis strains (n = 114) isolated in Casablanca. (PDF) [file pone.0047113.s003.pdf]

Supplemental Table S3. Allelic diversity of MIRU markers observed for *M. tuberculosis* strains (n=114) isolated in Casablanca.

MIRU-VNTR locus	Number of repeats								HGDI[a]
	1	2	3	4	5	6	7	8	
2	14	91	9						0.345
4	3	97	12	2					0.266
10			24	52	32	2		4	0.673
16	6	16	92						0.329
20	26	88							0.355
23	1	1	29	10	31	36	6		0.758
24	114								0
26	6	1	3	9	61	15	9	10	0.679
27	1	4	109						0.085
31		8	78	27	1				0.475
39	3	111							0.052
40	5	24	47	25	4	5	3	1	0.738

[a] HGDI, Hunter and Gaston Discriminatory Index.
